# An LC–MS/MS method for the determination of budesonide and 16α-hydroxyprednisolone in dog plasma

**DOI:** 10.1016/j.mex.2016.02.004

**Published:** 2016-02-24

**Authors:** Teresa Gazzotti, Andrea Barbarossa, Elisa Zironi, Paola Roncada, Marco Pietra, Giampiero Pagliuca

**Affiliations:** Department of Veterinary Medical Sciences – University of Bologna, Via Tolara di Sopra 50, 40064 Ozzano dell’Emilia (BO), Italy

**Keywords:** Budesonide, 16α-Hydroxyprednisolone, Plasma, Dog, LC–MS/MS

## Abstract

Although budesonide is frequently used in veterinary medicine for the treatment of canine respiratory and bowel inflammatory diseases, knowledge is lacking regarding its kinetics in this species. We developed and validated a liquid chromatography–tandem mass spectrometry method for the determination of budesonide and its metabolite 16α-hydroxyprednisolone in dog plasma. The analytes were extracted by solid phase extraction and analysis was performed by high performance liquid chromatography–tandem mass spectrometry, with positive electrospray ionization.

• This method allows budesonide and one of its main metabolites to be simultaneously quantified in dog plasma at fairly low concentrations.
• The proposed protocol is very easy and fast to execute, without compromising analytical performances.
• A small amount (0.5 mL) of plasma is required, making this approach suitable for pharmacokinetic studies also in small sized dogs.

## Graphical abstract

**Figure:**
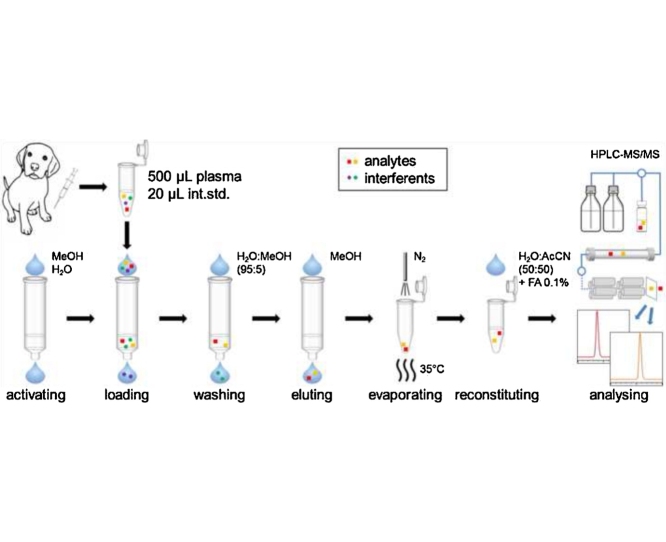

## Method details

An LC–MS/MS method for the quantitative determination of budesonide (BUD) and one of its main metabolite, 16α-hydroxyprednisolone (16α-HP), in dog plasma was developed and validated. The simultaneous quantification of the two molecules, the rapidity and simplicity of the procedure, and the low volume of sample required make the proposed technique suitable for pharmacokinetic studies involving also small-sized dogs. In addition, the developed protocol was employed in a clinical trial on dogs of different breeds, orally treated with BUD once a day for 30 days, demonstrating to be suitable for the purpose [1].

### Materials

BUD and betamethasone (BET) standards were purchased from Sigma–Aldrich (Milan, Italy); 16α-HP was purchased from Du-Hope International Group (Nanjing, China). The internal standard solution was prepared diluting BET in water:acetonitrile (1:1) at a concentration of 50 ng/mL.

### Sample preparation

1. Add 20 μL of BET internal standard solution to 0.5 mL of plasma.
2. Activate the Strata-X cartridge (1 mL, 30 mg) (Phenomenex, Torrance, USA) with 1 mL of methanol and wash with 1 mL of water.
3. Load the sample on the cartridge, at a flow speed of 2 mL/min.
4. Wash the cartridge with 3 mL of a solution of 5% methanol in water.
5. Dry the cartridge under low vacuum conditions for 30 s.
6. Elute the analytes with 1.5 mL of methanol at a flow speed of 2 mL/min, and completely remove the remaining solvent through vacuum.
7. Evaporate the eluate to dryness under nitrogen stream and heating at 35 °C.
8. Dissolve the sample in 100 μL of a mixture of water: acetonitrile (1:1) containing 1% formic acid and vortex for 30 s.
9. Transfer the reconstituted extract to autosampler vial for LC–MS/MS analysis.

### LC–MS/MS conditions

The liquid chromatograph was an Alliance 2695 system consisting of a quaternary pump, solvent degasser, auto sampler and column heater (Waters Corporation, Milford, USA). Chromatographic separation was achieved on a Waters XBridge MS C18 column (3.5 μm, 2.1 mm × 150 mm) in combination with a protecting guard column (3.5 μm, 2.1 mm × 5 mm) of the same type (Waters Corporation, Milford, USA). The mobile phase was water containing 0.1% formic acid (A) and acetonitrile containing 0.1% formic acid (B).

**Fig. 1:**
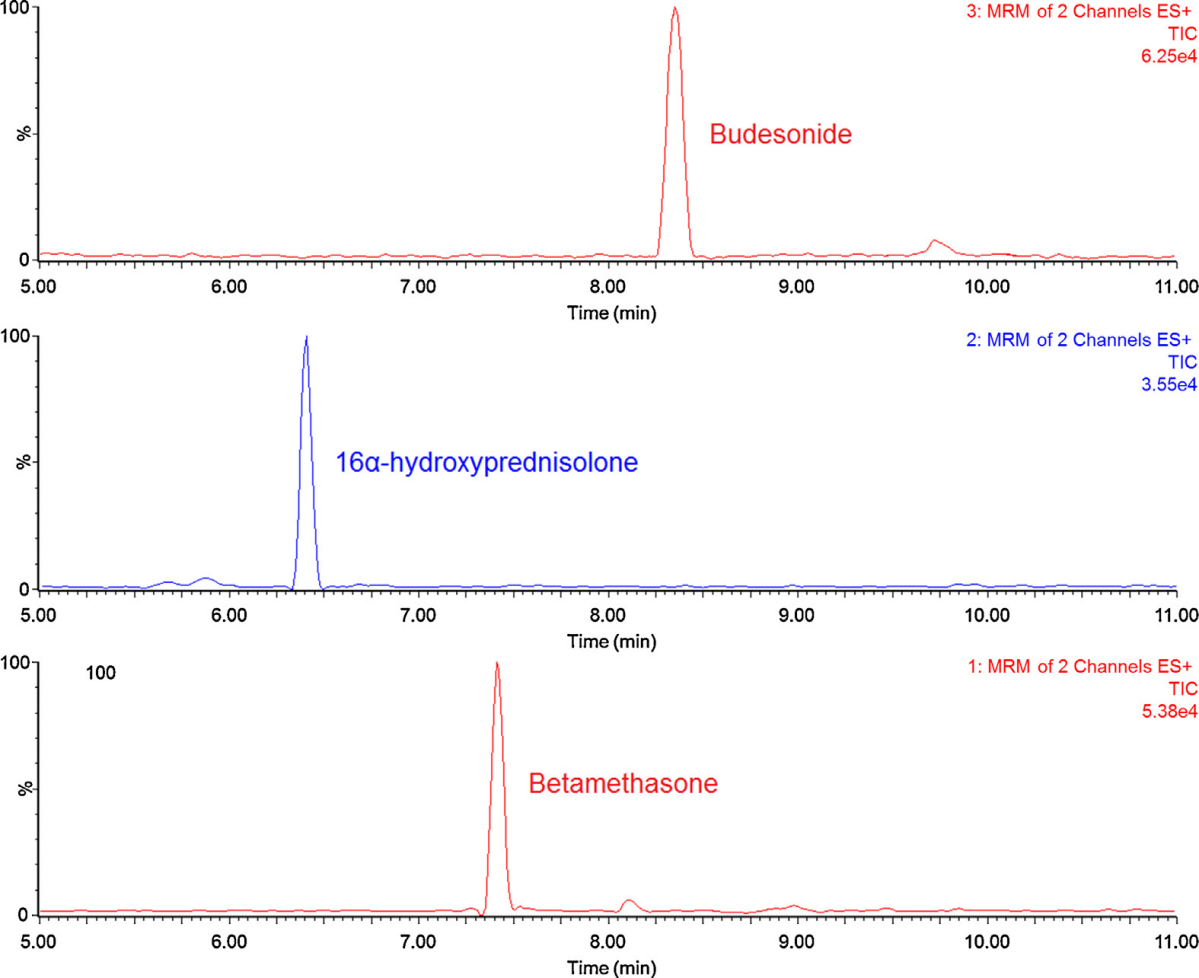
Chromatograms of a blank dog plasma sample fortified with budesonide (1.0 ng/mL), 16α-hydroxyprednisolone (1.0 ng/mL) and IS betamethasone (2.0 ng/mL).

The following gradient program, time (% A − % B), was applied: 0 min (90–10), 5 min (10–90), 8.5 min (10–90), 9.5 min (90–10), 13.0 min (90–10). The flow rate was 0.3 mL/min and the column temperature was maintained at 35 °C. An example of the obtained chromatograms is shown in Fig. 1.

Detection was carried out using a Quattro Premier XE triple quadrupole mass spectrometer equipped with an ESCI Multi-Mode Ionization Source (Waters Corporation, Milford, USA). The mass spectrometer operated in positive electrospray ionization mode (ESI+) with the following conditions: capillary voltage 3.25 kV; source and desolvation temperature 145 °C and 500 °C, respectively; desolvation and cone gas (nitrogen) flow 1000 L/h and 100 L/h, respectively. Acquisition was performed in SRM (Selected Reaction Monitoring) mode, monitoring two specific transitions for each compound, with a dwell time of 100 ms. The analyte-dependent MS/MS parameters were optimized via direct infusion of tuning standard solution into the mass spectrometer. The selected values of cone voltage, collision energy and the two main transitions monitored for each compound are given in Table 1. Data analysis was performed using MassLynx 4.1 Software (Waters Corporation, Milford, USA).

### Method validation

The proposed LC–MS/MS method was validated according to current European guidelines [2], taking into account specificity, linearity, accuracy, precision and limit of quantification (LOQ). The specificity was evaluated checking the ion chromatograms of blank samples collected from 21 dogs and analyzed for potential interfering compounds co-eluting at the specific retention times of the analytes.

The linearity of the method was assessed with five-points matrix-matched calibration curves, obtained by spiking pooled blank samples of plasma with standard solutions of BUD and 16α-HP at 0, 0.25, 0.5, 1, 5 and 10 ng/mL, and freshly prepared during different days. Peak area ratios between each analyte and BET (internal standard) were plotted against their concentration ratios, and a linear regression least square regression model was applied, showing a good linearity (*R*^2^ > 0.99).

The precision and accuracy of the assay were tested by analyzing blank samples fortified at three concentrations (0.5, 1 and 5 ng/mL) for both analytes. In particular, 18 spiked samples for each level were processed on three different days (six samples per day). The precision was measured as relative standard deviation to the mean (CV%) of both repeatability (within-day) and reproducibility (between-days). The results of the accuracy and precision experiments (Table 2) confirmed the good performances of the method.

The limit of quantification (LOQ) of the method, defined as the measured concentration with a signal to noise (S/N) ratio greater than 10, and acceptable precision and accuracy, was 0.25 ng/mL for both analytes.

The absence of matrix ion suppression was assessed by the post column infusion technique: during the injection of a blank matrix sample in the LC–MS system, BUD and 16α-HP standard solutions were directly and continuously infused into the MS interface [3].

## Additional information

### Background

Corticosteroids are anti-inflammatory drugs frequently used for the treatment of a wide range of diseases both in human and veterinary medicine. BUD is a non-halogenated glucocorticoid recently introduced in veterinary medicine for the treatment of respiratory and inflammatory bowel diseases (IBD), particularly in dogs refractory to systemic steroids. However, BUD veterinary-labeled products are not available on the market to date. BUD is rapidly metabolized and one of the main products in human is 16α-HP [2]. Although many different works have been published on the detection of BUD in human plasma [4], [5], [6], [7], only two studies are available on its determination in dog plasma, one of which written in Chinese [8], [9]; in addition, none of them simultaneously investigated both BUD and 16α-HP.

## Figures and Tables

**Table 1:** Retention times and mass spectrometric details of budesonide, 16α-hydroxyprednisolone and betamethasone (internal standard).

Analyte	Retention time (min)	Cone voltage (V)	Collision energy (eV)	Quantification transition (*m/z*)	Confirmation transition (*m/z*)
BUD	8.35	15	11	431 > 413	431 > 323
16α-HP	6.41	14	10	377 > 359	377 > 323
BET	7.42	16	8	393 > 373	393 > 147

**Table 2:** Results of method validation.

Analyte	Parameter	Fortification level		
		0.5 ng/mL	1 ng/mL	5 ng/mL
	Repeatability (CV%)	8.1	9.1	13.0
BUD	Reproducibility (CV%)	14.1	12.8	13.7
	Accuracy (bias%)	2.4	−2.6	−3.0

	Repeatability (CV%)	9.4	1.6	9.2
16α-HP	Reproducibility (CV%)	18.9	12.3	11.3
	Accuracy (bias%)	0.0	9.2	−5.3
